# Nanopatterning of Bionic Materials

**DOI:** 10.3390/nano13020233

**Published:** 2023-01-04

**Authors:** Johannes Heitz

**Affiliations:** Institute of Applied Physics, Johannes Kepler University Linz, Altenberger Strasse 69, 4040 Linz, Austria; johannes.heitz@jku.at; Tel.: +43-732-2468-9404

## Introduction

The nanopatterning of bionic materials, performed by means of laser processes that utilize pulsed laser sources with short and ultrashort pulse durations, is a rapidly growing field. The method’s applications are varied, being used mainly to tailor special industrial, medical, and scientific applications. This process is significantly driven by the exciting properties of micro- and nanopatterned materials found in natural biological species. These include being self-cleaning, capable of adapting color and reflectivity, the possession of pronounced adhesive and anti-adhesive properties, the aptitude for wetting and directional fluid transport, reduction of wear and friction, control of cell growth, and antimicrobiotic properties [1].

This Special Issue, entitled “Nanopatterning of Bionic Materials”, represents an extension of the former review and includes a collection of recent top-quality articles in this research area. Particular attention has been devoted to the functionalization of medical implants and other surfaces, relevant for biomedical applications owing to the widespread need for improved approaches in medicine in our aging society. A comparable scenario applies for miniaturized sensors concerning the sensitivity of small traces of biologically hazardous or metabolic gases.

Herein, only original research articles have been considered for publication. The present Special Issue includes contributions on the following research topics:

(i) Functionalization of Ti-based medical implants by means of laser-induced micro- and nanostructures for cell repellence as well as for osteoblastic proliferation and differentiation [2,3,4];
(ii) Polymer surfaces with antimicrobiotic and antiadhesive properties induced by laser-induced periodic surface structures (LIPSS) in connection with nanofibers or nanowires [5,6,7];
(iii) The generation of novel hybrid carbon nanotube materials for biosensing applications by means of laser-induced forward transfer (LIFT) [8].

Overall, I am grateful to all the authors for their fine contributions to the present Special Issue, and hope that the published studies will pave the way for the novel real-world applications of nanomaterials in medicine and biotechnology.
